# Social networks and the mental health among Chinese older adults: the mediating role of loneliness and moderating role of Internet use

**DOI:** 10.3389/fpubh.2023.1242356

**Published:** 2023-10-03

**Authors:** Jiayin Tian, Haohao Li

**Affiliations:** School of Sociology and Population Studies, Renmin University of China, Beijing, China

**Keywords:** social networks, mental health, loneliness, Internet use, older adults

## Abstract

**Background:**

Although a large body of research suggests that social networks from family and friends are important factors in protecting the mental health of older adults, we know little about the mediating and moderating mechanisms behind this relationship. Using China as an example, this study aims to investigate a comprehensive model that includes social networks, loneliness, Internet use, and mental health outcomes in the older population.

**Methods:**

We analyzed data from 7,648 Chinese older people over 60 using the 2018 CLASS survey. We studied how various social networks affect their mental health. Using SPSS’s PROCESS macro, we first employed descriptive statistics to examine the characteristics of the participants and calculate the correlations of core variables. Then, we assessed whether loneliness mediated this relationship and tested the moderated mediation effect of Internet use. Our findings shed light on these complex dynamics.

**Results:**

The statistics indicate a positive correlation between social networks and mental health. Furthermore, mediation models revealed that loneliness moderates the relationship between social networks and mental health. In addition, moderated mediation models revealed that Internet use played a distinct function in the family networks model compared to the friend networks model. Internet use moderates explicitly the effects of family networks on loneliness and friend networks on mental health.

**Conclusion:**

The findings emphasize the importance of differentiating the types of social networks to understand their impact on older adults well-being, encouraging policymakers, medical professionals, and families to adopt more targeted approaches when devising policy interventions and medical strategies, especially for older individuals with insufficient social support. Additionally, we urge governments to recognize the varying types of social networks among older populations and harness the protective effects of Internet technology on their well-being within a digital society.

## 1. Introduction

In recent years, population aging, mobility, and the Internet wave have been happening simultaneously in China (1–3). Since China entered the aging society in 2000, the speed of aging has been accelerating, and the scale of the older adult has been expanding. As a result, China has become the country with the largest older adults population in the world (2). Data from the National Bureau of Statistics show that by the end of 2022, the number of older adults aged 60 and above in China has reached 280 million, accounting for 19.8% of the total population. The older population tends to be more likely to face problems with their health than other age groups, and with mental health being an integral part of older people’s health (4), it is vital to understand how to maintain and improve the mental health of older adults.

Social networks are an important factor in maintaining and improving the mental health of older adults (5, 6). Social networks mainly include family and friend networks, and different types of networks play different roles in protecting older adults’ mental health (7). In China, blood ties are of great significance, and family networks in general, especially parent–child networks, are one of the most critical resources in the lives of older adults and the primary source of support for more aging parents (8). Thus, for older Chinese adults, the protective effect of family networks may be more robust, but it needs to be verified. According to social support theory, the protection of social networks for older adults’ mental health includes a main effect and a buffer effect. Some studies have shown that older adults’ social networks protect their mental health by reducing loneliness (9), which suggests that loneliness may moderate the relationship between social networks and older people’s mental health.

However, the social networks of older adults are under threat due to the increased mobility of the population in China’s modernization process. On the one hand, the outflow of young people and the retention of older people have disrupted older people’s family networks (10). On the other hand, the older adult may follow their children in migration and lose contact with their friends. Consequently, an increasing number of Chinese older adults face social isolation, threatening their mental health (11). Although impaired social networks may increase the risk of depression in older adults either directly or through increased loneliness, it appears that not all older adults feel lonely and suffer from depression when faced with an inadequate social network (12). According to the risk and resilience framework (13), this difference may be due to protective factors.

Along with the rise of the Internet wave in China, the number of older Internet users is increasing, with 42% of Chinese older adults using the Internet in 2020 (2). Given the increasing influence of the Internet, it is necessary to explore the impact of Internet use on the mental health of older adults. First, research has found that higher Internet use complements interpersonal contact, engagement, and community commitment. Thus, Internet use may be protective when traditional social networks are inadequate (2, 14). Second, online tools are also associated with perceived loneliness in older adults. The Internet can distract older adults from the experience of loneliness and reduce life stress by pulling them into the virtual world (15). Therefore, it is reasonable to assume that Internet use can moderate the direct and indirect links between social networks and older adults’ mental health.

In summary, while the existing literature indicates diverse associations between social networks, loneliness, mental health, and Internet use among older adults (16, 17), two critical research gaps remain unaddressed. First, fewer existing studies have delved into the heterogeneity of how different social networks affect mental health in older adults. Secondly, prevailing research has predominantly focused on examining the direct effects of the Internet on older people’s mental health (1, 18), overlooking the potential moderating role of the Internet in influencing the direct and indirect ramifications of social networks on mental health. In this study, we propose a moderated mediation model, hypothesizing that different types of social networks are associated with mental health among Chinese older adults, with loneliness playing a mediating role. It further posits disparities in these associations. Additionally, the study examines how Internet use moderates these direct and indirect relationships. By offering a more detailed analysis of the mechanisms through which social networks impact the mental health of older adults in the Internet era, this research aims to provide a theoretical foundation for guiding older adults’ use of the Internet and protecting their mental health.

### 1.1. The Main and mediating pathways

Social networks are an available resource for people to access various types of support (19, 20). Social support theory states that social networks can protect individuals’ physical and psychological health through direct and buffering effects (21). At the same time, many empirical studies have shown that social networks are closely related to the mental health of individuals; social networks play a protective role in the mental health of individuals, especially older adults (5, 22).

Social networks change over time and throughout an individual’s life course. However, there is no consensus on whether social networks change in old age compared to other age groups and their impact on mental health. One view is that older adults are more likely to have reduced social networks due to the stressful events they are likely to experience in old age, including widowhood, retirement, and illness (23) and that such reduced social networks may leave older adults in a state of social isolation and impair their mental health (24). Other studies have found that social networks remain stable in old age. For older adults, the quality of social networks is more important than quantity, and such long-lasting social relationships protect the mental health of older adults (9).

Social networks are multi-dimensional. According to the social convoy model theory, the two most important social networks for older adults are family and friend networks (19, 25). Family networks, consisting mainly of spouses and children, are located in the inner circle, and these networks are innate and determined by blood ties. Older adults have very stable relationships with these family members. Friend networks, formed by acquaintances, colleagues, and neighbors, are located in the outer circle (26), and friend networks rely on interactions between individuals and are relatively less stable (27).

There may be differences in the relationship between family and friend networks and older adults’ mental health. Socioemotional selectivity theory (SST) is the dominant theory in the emotional and social aging field, which can help us understand the significance of different social networks for older adults (28, 29). According to the theory, as people age, their motivational orientations shift as they perceive time to be limited. The perception of limited time activates goals related to emotional significance and influences motivational preferences. Older adults seek more emotion and meaning than younger people and particularly value family members who play an essential role in their lives. In China, a similar theory has been proposed called “Cha Xu Ge Ju” (30). This theory suggests that in China, blood ties are important, and social networks are centered on the individual, followed by immediate family, other relatives, neighborhood, and friends, with the further away from the individual, the lower the importance.

Based on the above theoretical and literature analysis, we propose hypothesis 1:

*Hypothesis 1*: There is a positive association between social networks and the mental health of older Chinese people. Compared to friend networks, The correlation is more robust for family networks.

The pathways through which social network dimensions influence the mental health of older adults remain unknown. Research suggests that loneliness negatively predicts mental health. Individuals experiencing loneliness tend to have poorer mental health (16, 31, 32). Loneliness is a subjective feeling often characterized by a sense of isolation, a lack of social belonging, and an unpleasant intimate experience (33, 34). It has also been conceptualized as distress arising from discrepancies between an individual’s desired and actual social relationships (35). Among the factors influencing loneliness, the role of social networks is significant. Inadequate social networks positively predict feelings of loneliness (5, 36). Kovacs et al. (37) found that individuals with less than five close confidants during a crisis were likelier to report enhanced loneliness (37). The above studies suggest that loneliness may mediate the relationship between social networks and mental health and act as a mental health risk factor in older adults.

Some studies have supported the social networks→loneliness→mental health pathway. Qian’s (38) study found that for people living with HIV, loneliness mediates the decline in mental health due to social support (38). Park et al. (9) studied 209 older Korean adults in Central Texas. They found that older adults were at an increased risk of poor mental health when experiencing a severe lack of social connections, also known as social isolation. Further mechanistic studies revealed that loneliness mediated the relationship between social isolation and depressive symptoms (9). However, it remains unclear whether loneliness also mediates the relationship between family/friend networks and mental health among older Chinese individuals.

Based on the above theoretical and literature analysis, we propose hypothesis 2:

*Hypothesis 2*: Loneliness mediates the relationship between family/friend networks and older adults’ mental health.

### 1.2. The moderating role of Internet use

The widespread availability of Internet installations in China coincides with the increasing trend of “Netizens” aged 60 and older. Internet use has become an indispensable lifestyle for the older Chinese and is crucial for their healthy psychological development (39). The emergence and widespread use of the Internet have led scholars to concentrate on this new element. A systematic review has determined that Internet use can be a protective factor during an individual’s positive development (40). Theoretically, Internet use can free older people from stigma and limited Internal ability as a new form of social engagement (41, 42). Many studies have revealed that Internet use has a significant impact on reducing loneliness and depression, as well as on improving social support among older people (1).

From social capital theory, social capital refers broadly to the resources that emerge from one’s social ties (43, 44) and play a significant role in determining self-rated health, healthy behavior, and mental health (45). Studies have found that Internet use buffers the negative link between age and social capital, acting as a priceless tool for preserving existing friendships, enhancing familial bonds, and fostering virtual connections (46). Bonding and bridging social capital are the two main components of social capital (44). On the one hand, older adults who have been using the Internet for a long time can break time and space restrictions and use Internet devices to maintain close contact with family and friends (known as “bonding social capital”), which enables them to reach out for assistance more easily and quickly. Therefore, Internet use by senior individuals is more conducive to the protective effect of their social networks on mental health, especially for older people with disabilities (47). On the other hand, virtual networks facilitate the expansion and diversification of social ties between various generations and social groups (48). Using the Internet broadens older people’s exposure to more resources. It helps them meet more people from varied backgrounds (known as “bridging social capital”) (49, 50), which will effectively mitigate the negative effects of low family/friend networks. The benefits mentioned above of Internet use might increase the positive impact and reduce the negative impact of social networks on older people.

Moreover, Internet use can also impact the role of loneliness as a mechanism between social networks and mental health, and a wide range of Internet use interventions have been tested to reduce loneliness among older adults (51). From the definition of loneliness, researchers have deduced that three coping strategies can alleviate loneliness and its negative effects: building supportive networks, lowering standards, and putting loneliness into perspective (15). First, the Internet has shown some potential to benefit older adults regarding their supportive networks by enhancing personality traits like self-confidence, perceived self-efficacy, and perceived control (52) and reducing the adverse effects of age-related stereotypes (53). Previous studies have found that even with the same family and friend networks, increased Internet use supplements interpersonal contact, engagement, and community commitment, providing individuals access to more supportive networking and potentially reducing feelings of loneliness (17). Secondly, using Internet technologies is connected with putting loneliness into perspective among older adults. The Internet could distract older adults from their loneliness experience and lower perceived life stress by pulling them into virtual worlds (54). Consequently, higher Internet use may also mitigate the negative effects of loneliness. Therefore, Internet use may play a risk-buffer role and alleviate the adverse effects of loneliness on the mental health of older adults. Based on the findings of previous research, We formulate hypothesis 3:

*Hypothesis 3*: Internet use moderated direct and indirect relationships between family/friend networks and older adults’ mental health. Specifically, Internet use would buffer the direct effect of family/friend networks on mental health (Hypothesis 3a) and buffer the mediating influence of loneliness on the effect of family/friend networks on mental health (Hypothesis 3b).

**Figure 1:**
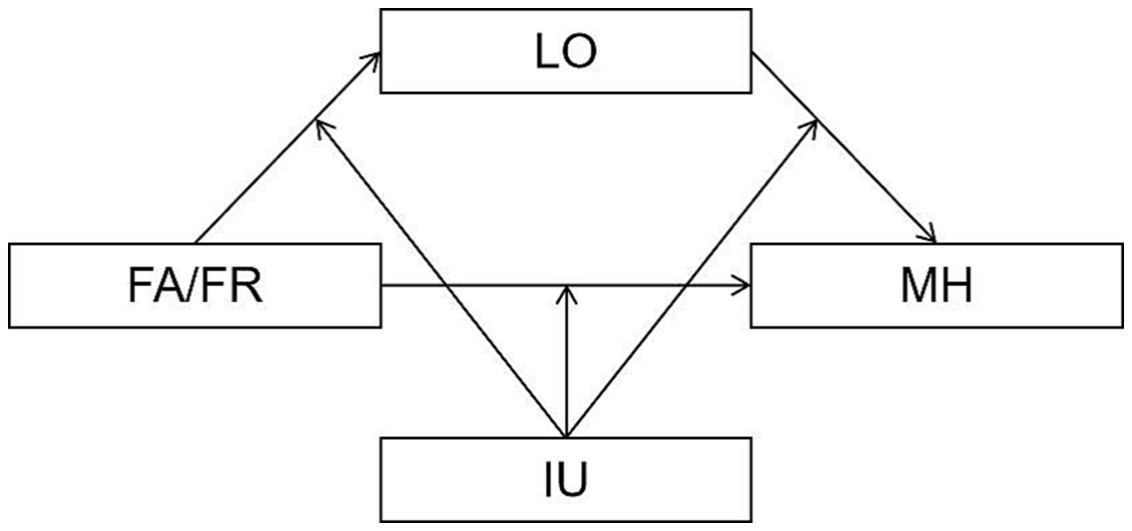
The proposed mediated moderation model. FA, Family networks; FR, Friend networks; LO, loneliness; MH, Mental health.

This study’s primary objectives were to explore the relationships between Social networks (SN) and Mental health (MH) and to test whether the influence of SN on MH is mediated by loneliness (LO) and moderated by Internet use (IU). Based on the results of previous studies, Figure 1 illustrates three hypotheses.

## 2. Materials and methods

### 2.1. Data source

The data used in this study are from the 2018 waves of China Longitudinal Aging Social Survey (CLASS). CLASS is a nationally representative panel study that has followed adults aged 60 and above in China at 2-year intervals since 2014(data and documentation available at http://class.ruc.edu.cn). CLASS aims to collect plentiful information about health measures, family relationships, and social participation from approximately 11,000 Chinese respondents over 60 using a stratified multi-stage probability sampling method. In 2018, we surveyed 11,418 participants; only those who replied properly to at least three of the cognitive questions in the CLASS survey were eligible to proceed to the mental health questions. Consequently, the respondents’ cognitive abilities influenced the data for key variables such as mental health and loneliness, resulting in many missing values (*N* = 2,184). Additionally, due to the higher sensitivity of the personal income question, 1,405 (12.3%) respondents selected options such as “do not know” or “do not want to answer.” Furthermore, the gender distribution (male, 50.34%) is almost identical to that of the full sample (male, 50.24%), and the mean age of the final sample (Mage = 71.2) is approximately the same as that of the full sample (Mage = 71.4), which indicates a good representation. After excluding observations with missing values for the variable we are interested in, 7,648 valid observations were finally included in the analysis. The final samples consisted of 3,850 males (Mage = 71.13, SDage =7.15) and 3,798 females (Mage =71.29, SDage =7.37).

### 2.2. Variable design

#### 2.2.1. Explained variables

The dependent variable in this study was mental health. The 9-item Depression Scale (CES-D) was used to measure mental health according to the commonly used measures of mental health (55). We asked the participants about the frequency of depressive symptoms during the past week. We assessed responses to each question using a 3-point Likert scale, where “1” means “hardly ever or never” and “3” means “often.” We reverse-recorded six negative items and summed the result after reverse coding as a continuous variable, with higher values indicating better mental health. This method has been validated in previous studies worldwide (56, 57), with Cronbach’s alpha coefficient of the scale being 0.71.

#### 2.2.2. Independent variables

The independent variable was the social networks of older adults, divided into family and friend networks. We used the Lubben Social Networks Scale (LSNS), a reliable and valid instrument to measure the quality, intimacy, and frequency of participants’ social relationships (58) to measure the social networks of older Chinese adults (59). The scale comprises six questions:(1) How many relatives/friends do you see or talk to at least once a month? (2) How many relatives/friends do you feel more comfortable talking to about personal matters? (3) How many relatives/friends do you feel close enough to ask for help? We coded responses to each question as (0 = none, 1 = 1 person, 2 = 2 persons, 3 = 3 or 4 persons, 4 = 5 to 8 persons, and 5 = 9 or more persons). The final scores for both family and friend networks were on a scale of 0–15. Higher scores mean that older people have closer family and friend relationships. The Cronbach’s coefficient was 0.82 for the family network scale and 0.84 for the friend network scale.

#### 2.2.3. Mediating variables

The mediating variable was loneliness in this study. We use the UCLA loneliness scale to assess loneliness. The scale comprises three questions: (1) How frequently do you experience a lack of social interaction? (2) How frequently do you feel disconnected from the individuals around you? and (3) How frequently do you feel alienated from others? A 3-point Likert scale, with “1” meaning “rarely” and “3” meaning “most of the time,” was used to evaluate each response in every case. Responses were assessed on a 5-point Likert scale, with “1” indicating “never,” “2” indicating “rarely,” “3” indicating “sometimes,” “4” indicating “often,” and “5” indicating “always.” The validity and reliability of this scale have been tested in a previous study (60). Reliability analyzes revealed that Cronbach’s alpha coefficient of the scale was 0.77.

#### 2.2.4. Moderating variables

The moderating variable in this study was Internet use. Following previous research, We use a signaling question to measure Internet use (“How frequently do you access the Internet?). This measurement method has been widely used in studies worldwide as a quick measure of older adults Internet use (18, 61).

#### 2.2.5. Control variables

Literature reveals that the mental health of older adults has numerous correlations and varies by socio-demographic background, health status, and social environment (30, 62). To control potential effects, We use socio-demographic characteristics as control factors, including age (continuous variable), gender (male = 1), living area (urban = 1), marital status (widowed = 1), educational level (0 = less than elementary; 1 = elementary; 2 = secondary; 3 = college and above), self-rated health (poor to excellent), functional health (ADL), income (continuous variable), and the number of children (continuous variable).

### 2.3. Analytical strategy

First, we used descriptive statistics to examine the characteristics of the participants and calculate the correlations of core variables. Second, we used bootstrapping to test whether loneliness mediates the effect of social networks on older adults’ mental health. Finally, we added Internet use to the mediation model to test the moderated mediation effect.

We use IBM SPSS Statistics (version 24.0) to conduct statistical analysis. We use the PROCESS macro (version 4.1) to analyze the mediating role of loneliness and the moderating role of Internet use. The bootstrapping method produces 95% bias-corrected confidence intervals (CIs) for these effects from 5,000 bootstraps resamples. CIs (95%) without zero indicate a significant effect.

## 3. Results

### 3.1. Descriptive statistics

Table 1 shows the socio-demographic characteristics of older adults and the statistics of all variables. The average age of the respondents was 71.21 years; 49.66% were male, 60.33% lived in rural areas, 70.24% had a spouse, and 63.38% were educated below middle school. The average annual income(logged) of the respondents was 8.19 (SD = 1.43), the mean score of activity of daily living (ADL) was 6.21 (SD = 1.01), the mean score of self-assessed health was 0.68 (SD = 0.71). The average family networks scale score was 7.37 (SD = 2.78), the average friend networks scale score was 6.41 (SD = 3.17), the average loneliness scale score was 4.46 (SD = 1.57), the average Internet use score was 1.74 (SD = 1.50). The average mental health scale score was 18.73 (SD = 2.63).

**Table 1:** Descriptive statistics.

Variables	Mean	SD	Min	Max
Age	71.21	7.26	60	100
Gender				
Female (*n*, %)	3,850	50.34		
Male (*n*, %)	3,798	49.66		
Residence				
Urban (*n*, %)	3,034	39.67		
Rural (*n*, %)	4,614	60.33		
Marriage				
Married (*n*, %)	5,372	70.24		
Unpartnered (*n*, %)	2,276	29.76		
Education				
Lower than middle school (*n*, %)	4,847	63.38		
Middle school and above (*n*, %)	2,801	36.62		
Income (logged)	8.19	1.43	4.09	16.12
ADL	6.21	1.01	6	18
Self-assessed health	0.68	0.71	0	2
Family networks	7.37	2.78	0	15
Friend networks	6.41	3.17	0	15
Loneliness	4.46	1.57	3	9
Internet use	1.74	1.50	1	5
Mental health	18.73	2.63	9	24

*N* = 7,648, SD, standard deviation.

Table 2 shows the correlations between the main variables. Family networks, friend networks, and loneliness were all significantly associated with the mental health of older adults(r = 0.110, *p* < 0.001;r = 0.113, *p* < 0.001; r = −0.489, *p* < 0.001), and both family networks and friend networks were significantly associated with loneliness(r = −0.144, *p* < 0.001; r = −0.091, *p* < 0.001), suggesting the potential role of loneliness in mediating the relationship between different types of social networks, and the mental health of older adults. In addition, Internet use was significantly associated with family networks(r = 0.082, *p* < 0.001) and friend networks(r = 0.118, *p* < 0.001) and loneliness(r = −0.134, *p* < 0.001) and mental health(r = 0.135, *p* < 0.001), suggesting a potential role for this variable in modifying the relationship between different types of social loneliness, and mental health in older adults.

**Table 2:** Descriptive statistics and correlations of core variables.

	1	2	3	4	5
Family networks	–				
Friend networks	0.647***	–			
Loneliness	−0.144***	−0.091***	–		
Internet use	0.082***	0.118***	−0.134***	–	
Mental health	0.110***	0.113***	−0.489***	0.135***	–
ME	7.37	6.41	4.46	1.74	18.73
SD	2.78	3.17	1.57	1.50	2.63

*N* = 7,648, *p < 0.05, **p < 0.01, ***p < 0.001.

### 3.2. Analysis of primary and mediating effects

**Figure 2:**
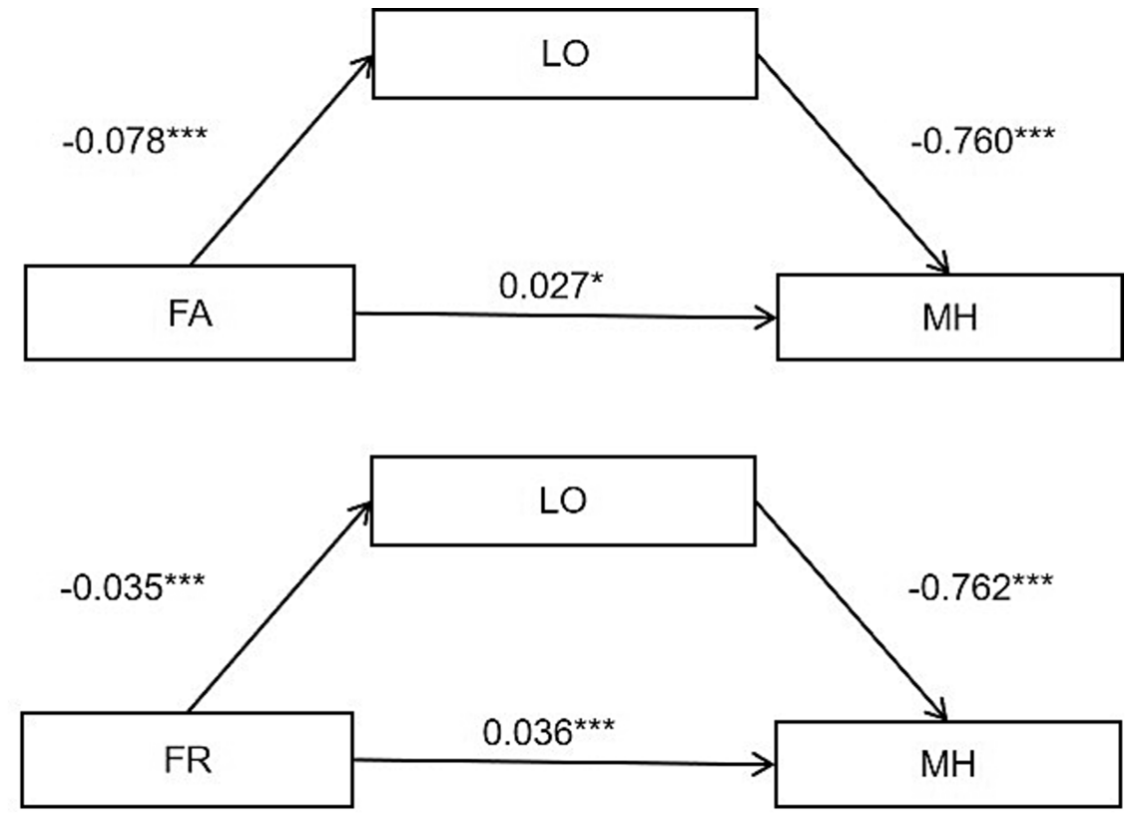
The mediating effect of loneliness on family networks/friend networks and mental health, *p < 0.05; **p < 0.01; ***p < 0.001.

To examine the relationship between family networks, friend networks, and older people’s mental health and whether loneliness plays a mediating role, we used SPSS 24.0 software, Process Model 4. Table 3 and Figure 2 show the results.

**Table 3:** Test results of main and mediating effect analysis.

Model pathways	*β*	SE	95% CI	
			Lower	Upper
Direct effects				
FA → MH	0.027*	0.009	0.009	0.045
FR → MH	0.036***	0.008	0.020	0.052
Indirect effects				
FA → LO → MH	0.059***	0.005	0.049	0.069
FR → LO → MH	0.027***	0.005	0.018	0.036
FA Total effect	0.086***	0.001	0.066	0.107
FR Total effect	0.063***	0.010	0.045	0.081

*N* = 7,648, *p < 0.05, **p < 0.01, ***p < 0.001.

First, the results in Table 3 show that family and friend networks are positively related to the mental health of older adults. The total effect of family networks is 0.086 (*p* < 0.001), the total effect of friend networks is 0.063(p < 0.001), and the impact of family networks is greater than that of friend networks. We proved hypothesis 1.

The results in Table 3 and Figure 2 indicate that family networks were positively associated with the mental health of older adults through two pathways. One is the direct effect, where the more robust the family networks, the higher the mental health of older adults (β = 0.027, *p* < 0.05). One is the indirect effect, in which the family networks negatively affect loneliness (β = −0.078, *p* < 0.078), which in turn affects the mental health of older adults (β = −0.078, *p* < 0.078), with the direct effect accounting for 31% and the indirect effect accounting for 69% of the total effect. Also, there is a mediating effect of loneliness in the relationship between the friend networks and the mental health of older adults, with the direct effect accounting for 57% and the indirect effect accounting for 43% of the total effect. Hypothesis 2 was confirmed.

### 3.3. Testing for the moderated mediation

To examine the moderating effects of Internet use on the direct and indirect effects of different types of social networks on the mental health of older adults, we used SPSS 24.0 software, Process Model 59. Table 4 shows the results.

**Table 4:** The moderated mediation model with loneliness as a mediator and Internet use as a moderator.

Variable	*β*	SE	95% CI	
			Lower	Upper
Loneliness				
FA × IU	0.010*	0.004	0.002	0.019
FR × IU	0.006	0.004	−0.002	0.014
Mental health				
FA × IU	−0.010	0.012	−0.034	0.014
FR × IU	−0.019***	0.006	−0.030	−0.008
LO × IU(FA)	−0.002	0.007	−0.015	0.011
LO × IU(FR)	−0.012	0.012	−0.036	0.011

*p < 0.05; **p < 0.01; ***p < 0.001.

Among the three moderated mediation models of family networks and the mental health of older adults, one model has detected a significant moderating effect (Table 4). The results showed that Internet use significantly regulated the relationship between family networks and loneliness (β = 0.010, SE = 0.004, LLCI = 0.002, ULCI = 0.019). Among the three moderated mediation models between friend networks and older adults mental health, one model also detected a significant moderating effect. The results showed that Internet use significantly moderated the relationship between friend networks and mental health (β = −0.019, SE = 0.006, LLCI = −0.030, ULCI = −0.008).

**Figure 3:**
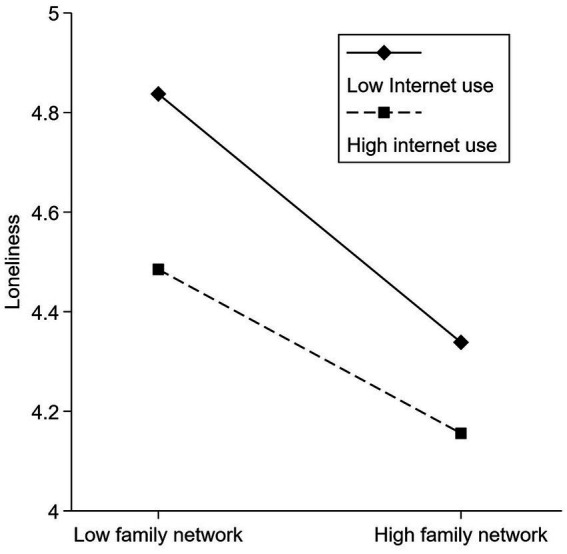
The moderating effect of Internet use on the relation between family networks and loneliness.

**Figure 4:**
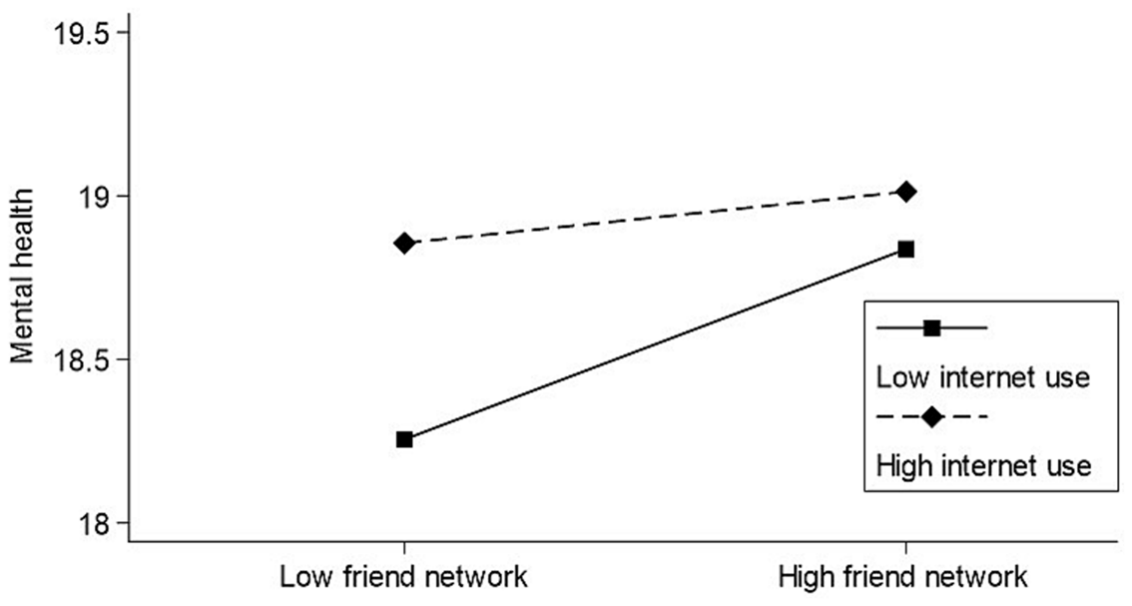
The moderating effect of Internet use on the relation between friend networks and mental health.

We constructed a moderating effect graph and further analyzed the moderating effect of Internet usage. Figure 3 shows that Internet use moderates the negative correlation between family networks and loneliness. The negative correlation will be weaker when older adults have high levels of Internet use. Figure 4 shows that Internet use moderates a positive correlation between friend networks and the mental health of older adults. The positive correlation will be weaker when older adults have high levels of Internet use.

## 4. Discussion

In this study, we explored the relationship between different dimensions of social networks, including family and friend networks, and the mental health of older Chinese adults. In addition, we explored the mechanisms by which social networks affect the mental health of older adults through mediation analysis. Finally, we discuss the role that frequency of Internet use plays in the pathways by which social networks influence mental health in older adults. Both family and friend networks were positively associated with older people’s mental health in China, with the results indicating that higher-scoring social networks, especially family networks, are a significant component in protecting older people’s mental health. Loneliness is an important pathway through which family/friend networks affect older people’s mental health. Internet use moderates the indirect mechanism by which family networks affect older people’s mental health, and high Internet use frequency weakens the effect of family networks on older people’s loneliness. Internet use directly moderates the relationship between friend networks and older people’s mental health, and for older people with low friend networks, high Internet use frequency weakens the impairment of low friend networks on mental health.

### 4.1. Relationship between SN and MH

Overall, our first hypothesis was supported. That is, higher family/friend network scores are associated with higher levels of mental health. Similar studies have also demonstrated the association between social networks and mental health (26, 37, 63). However, our findings enrich and go beyond existing studies in two main ways: First, we focus on the 60+ older adults population in China, which is very large, given the aging of the Chinese population (6, 64). However, existing studies still inadequately address older Chinese adults’ social networks and mental health. Second, our study distinguished between family networks and friend networks, and the results showed that the relationship between family networks and their mental health was stronger among older Chinese adults than between friend networks. This study found that family networks have a more significant impact on older Chinese adults’ mental health than friend networks, consistent with the socioemotional selectivity theory and the Chinese “Cha Xu Ge Ju” theory. According to the socioemotional selectivity theory, older adults prioritize emotionally meaningful relationships, especially those with family members (29). From this perspective, decreasing social relationships in later life is a self-initiated process driven by prioritizing emotionally meaningful relationships over more extensive social networks (65). This result suggests that the quality of social networks is even more crucial for the social networks of older adults. Family networks, in particular, are vital for protecting the mental health of older adults. According to the “Cha Xu Ge Ju” theory, Chinese social relationships follow a self-centered differential pattern, including family and friend networks. However, these relationships have different statuses and affinity, and family networks, represented by blood ties, tend to be more important to Chinese people (6, 66). These effects suggest that different types of social networks have different utilities in protecting the mental health of Chinese older adults, with family networks being particularly significant.

### 4.2. The mediating role of LO

Furthermore, the findings suggest that higher-scoring family and friend networks are indirectly associated with mental health by reducing loneliness, in addition to being directly associated with mental health in older adults. Higher scores in family and friend networks may reflect the higher social capital possessed by older adults, and social capital may promote social interaction and participation (34, 38), reducing loneliness and promoting mental health in older adults. Previous studies have found similar findings (9, 63).

Another interesting finding is that in the relationship between family networks and older adults’ mental health, the indirect effect dominates, and the direct effect is relatively small, suggesting that family networks mainly affect older adults’ mental health by influencing their feelings of loneliness. In contrast, the direct effect dominated the relationship between friend networks and older adults’ mental health, suggesting that friend networks are more directly related to older adults’ mental health. This finding may echo some previous findings that the structure of social networks is related to loneliness, with social networks divided into close networks (family networks) and non-close networks (friend networks). Having more confidants or very close ties in one’s networks is usually associated with lower levels of loneliness (32, 67), while non-close networks, while also associated with lower levels of loneliness, are less protective (37, 68, 69).

Overall, loneliness mediates the relationship between family and friend networks, which verifies hypothesis 2. Also, loneliness is a risk factor for mental health in older adults, and we found that having closer family networks compared to friend networks is vital to reducing loneliness in older adults.

### 4.3. The moderating role of IU

The use of the Internet has a profound impact on the daily lives of older people, and it can affect their well-being by influencing how easily they can access social support from family and friends. Few studies, however, have examined how family/friend networks and loneliness interact to improve the mental health of older adults. The moderated mediation model found a more complex interaction effect among family/friend networks, loneliness, and mental health, with their relationships varying according to the frequency of Internet use. These findings are consistent with previous studies (70) showing that elders’ participation in virtual worlds is crucial to mental health.

Specifically, the moderating role of the Internet differed in the family and friend networks models. First, the frequency of Internet use moderates the negative relationship between family networks and loneliness, with older adults who used the Internet more frequently showing a weaker relationship than those who used it less frequently. It suggests that Internet use protects, as expected, against increased loneliness due to lower levels of family networks. This buffering effect supports the “stimulus hypothesis” that older people benefit from the social (71) and entertainment functions (72) of the Internet. They have more options for connecting with others (2) and more leisure activities (73). They are less concerned about loneliness in the online community (74), thus reducing the loneliness experience of low family networks. At the same time, this finding also suggests that Internet use protects against loneliness among older adults, even when they have strong family ties. It also fits with previous research highlighting how the older adults use ICTs to enhance their social interactions. This is achieved by overcoming geographical barriers and capitalizing on optimization strategies to augment their opportunities for connectivity (75). However, Internet use did not moderate the direct relationship between family networks and mental health, possibly because low levels of family networks were directly associated with increased depressive symptoms. Internet use could provide older people access to a broader range of individuals from different backgrounds, bringing in more bridging social capital than family/friend networks (76). Thus, Internet use and family networks may affect older people’s mental health separately. In future studies, We can further insight into this finding through different qualitative research methods, such as group interviews.

The frequency of Internet use moderated the positive relationship between friend networks and mental health, and this relationship appeared to be weaker for older adults who used the Internet more frequently than for those who used it less frequently. One possible explanation is that the Internet has become a window into the world for older people, increasing the importance of their enjoyment of life and allowing them to learn about and have a more interesting life (64). The Internet also increases their perception of the social capital associated with friends and the likelihood of rekindling previous relationships (e.g., high school friends) (77). Thus, this incremental finding is consistent with previous research showing that the Internet can amplify mental health outcomes through increased friend networks. Nevertheless, unlike the family networks model, Internet use has not significantly changed the relationship between friend networks and loneliness. This result may be explained by the fact that the social relationships of older Chinese people are mainly in a “Cha Xu Ge Ju “model, and their experience of loneliness is more directly related to their family networks. At the same time, older people’s friendships are stable, and they are more concerned with the quality than the quantity of their friendships, preferring face-to-face interactions (78, 79). They will only choose communication technology to stay in touch with close companions unless a long-distance relocation event occurs in later life (80). Additionally, Internet use did not moderate the relationship between loneliness and mental health, which may be directly related to loneliness and older people’s mental health.

Overall, the Internet moderated the negative relationship between family networks and loneliness and the positive relationship between friend networks and mental health. The more frequent Internet use predicted a higher level of mental health status, which partially verified Hypothesis 3. Also, the moderating role of the Internet differed in the family and friend networks models. The study found that encouraging online engagement effectively affected older people’s social networks, loneliness, and psychological well-being.

### 4.4. Limitations and implications

In conclusion, social network factors may contribute to a better understanding older Chinese adults’ mental health. The cross-sectional research methodology, however, restricted the interpretation of the causal association between social networks, loneliness, and mental health. Also, although this study was analyzed using control variables such as gender and age, future research should consider other confounding factors such as family features and socioeconomic status. These variables may influence the social participation and negative emotions of older adults. In addition, we used retrospective and self-reported measures to assess study variables, which are susceptible to common biases. Future studies should use a longitudinal design and control for possible confounders to determine causal associations and investigate the underlying mechanisms. Despite these limitations, this study is relevant as it suggests specific treatments for older Chinese adults, considering this population’s relatively low levels of mental health. Furthermore, an empirical basis for improving mental health levels in the Internet age is proposed using nationally representative data.

In summary, the results of our study have several immediate practical implications for the prevention and intervention of elders’ mental health. Firstly, given that social networks negatively predict loneliness and positively predict mental health, increasing the quantity and quality of family and friend networks may be an effective way to improve older people’s mental health. Secondly, this study suggests that loneliness may mediate between social networks and older people’s mental health. This indicates that identifying and focusing on older adults who experience more loneliness may help us improve the effectiveness of mental health interventions for older adults. Third, our study suggests that the Internet may help protect older adults from the loneliness and depression associated with social support networks. Therefore, encouraging older adults to use more Internet services, experiment with more Internet features, and increase their online capital could reduce loneliness and improve mental health.

More importantly, there are differences in the roles of family and friend networks. Family members are more significant to Chinese older adults and have a greater influence on their mental health. Internet use often weakens the negative relationship between family networks and loneliness while enhancing the positive relationship between friend networks and mental health. According to the findings of this study, policymakers, medical professionals, and families should be aware of the potential effects that might be caused by different types of networks and the various functions that the Internet can perform. Increased awareness could encourage more targeted improvements in policy interventions and medical interventions. It is necessary to focus on older people with inadequate family support, distinguish between different social networks for older people, and maximize the protective role of Internet technology.

## 5. Conclusion

This study tested a moderated mediation model to examine the psychological factors in the relationship between family/friend networks (FA/FR) and mental health (MH) in older Chinese adults. In short, the research revealed that social networks protected older Chinese adults’ mental health and predicted mental health by influencing loneliness (LO). In the family networks model, the effect of social networks on loneliness was buffered by Internet use. In contrast, the effect of friend networks on mental health was buffered by Internet use. These results significantly advance our understanding of older Chinese people’s mental health and social networks in the Internet age.

## Data availability statement

The original contributions presented in the study are included in the article/supplementary material, further inquiries can be directed to the corresponding author.

## Author contributions

HL and JT collected the data and performed the data analyzes, drafted and revised the manuscript. All authors contributed to the article and approved the submitted version.

## References

[1] Li L Jin G Guo Y Zhang Y Jing R. Internet access, support, usage divides, and depressive symptoms among older adults in China: a nationally representative cross-sectional study. J Affect Disord. (2023) 323:514–23. doi: 10.1016/j.jad.2022.12.001 36496102

[2] Tang D Jin YA Zhang K Wang DH. Internet use, social networks, and loneliness among the older population in China. Front Psychol. (2022) 13:895141. doi: 10.3389/fpsyg.2022.895141, PMID: 35645921 PMC9133735

[3] Aparicio SL Duarte I Castro L Nunes R. Equity in the Access of Chinese Immigrants to Healthcare Services in Portugal. Int J Environ Res Public Health. (2023) 20:442. doi: 10.3390/ijerph20032442 PMC9916350 36767820

[4] Steptoe A Deaton A Stone AA. Subjective wellbeing, health, and ageing. The Lancet. (2015) 385:640–8. doi: 10.1016/S0140-6736(13)61489-0 PMC4339610 25468152

[5] Bareket-Bojmel L Shahar G Abu-Kaf S Margalit M. Perceived social support, loneliness, and hope during the COVID-19 pandemic: testing a mediating model in the UK, USA, and Israel. Br J Clin Psychol. (2021) 60:133–48. doi: 10.1111/bjc.12285, PMID: 33624294 PMC8013849

[6] Tang B Mamubieke M Jilili M Liu L Yang B. Amelioration and deterioration: social network typologies and mental health among female domestic workers in China. Front Public Health. (2022) 10:899322. doi: 10.3389/fpubh.2022.899322, PMID: 36159277 PMC9492937

[7] Fuller HR Ajrouch KJ Antonucci TC. Original Voices The Convoy Model and Later-Life Family Relationships. J Fam Theory Rev. (2015) 12:126–46. doi: 10.1111/jftr.12376 PMC7283809 32536976

[8] Li T Zhang YL. Social network types and the health of older adults: exploring reciprocal associations. Soc Sci Med. (2015) 130:59–68. doi: 10.1016/j.socscimed.2015.02.007, PMID: 25681715

[9] Park N Jang Y Lee B Chiriboga DA. The relationship of living alone and depressive symptoms in older Korean Americans: the mediating role of loneliness. Gerontologist. (2015) 55:261–1. doi: 10.1093/geront/gnv569.18

[10] Fu Z Hao L. Agent-based modeling of China’s rural-urban migration and social network structure. Physica A. (2018) 490:1061–75. doi: 10.1016/j.physa.2017.08.145, PMID: 29200605 PMC5708571

[11] Sun JJ Jiang WK Li HH. Social isolation and loneliness among Chinese older adults: examining aging attitudes as mediators and moderators. Front Psychol. (2022) 13:1043921. doi: 10.3389/fpsyg.2022.1043921, PMID: 36562076 PMC9763440

[12] Valtorta NK Moore DC Barron L Stow D Hanratty B. Older Adults’ social relationships and health care utilization: a systematic review. Am J Public Health. (2018) 108:E1–E10. doi: 10.2105/Ajph.2017.304256, PMID: 29470115 PMC5844393

[13] Sameroff AJ. Vulnerable but invincible - a longitudinal-study of resilient children and youth-Werner, Ee, Smith. Rs Contemporary Psychol. (1983) 28:11–2. doi: 10.1037/021523

[14] Machesney D. Wexler S. S. Chen T. Coppola J. F. (2014). Gerontechnology companion: Virutal pets for dementia patients. 2014 Ieee Long Island Systems, Applications and Technology Conference (Lisat).

[15] Fokkema T Knipscheer K. Escape loneliness by going digital: a quantitative and qualitative evaluation of a Dutch experiment in using ECT to overcome loneliness among older adults. Aging Ment Health. (2007) 11:496–504. doi: 10.1080/13607860701366129, PMID: 17882587

[16] Rico-Uribe LA Caballero FF Olaya B Tobiasz-Adamczyk B Koskinen S Leonardi M. Loneliness, social networks, and health: a cross-sectional study in three countries. PLoS One. (2016) 11:e0145264. doi: 10.1371/journal.pone.0145264, PMID: 26761205 PMC4711964

[17] Wellman B Quan Haase A Witte J Hampton K. Does the internet increase, decrease, or supplement social capital? Social networks, participation, and community commitment. Am Behav Sci. (2001) 45:436–55. doi: 10.1177/00027640121957286

[18] Zhang H Wang H Yan H Wang X. Impact of internet use on mental health among elderly individuals: a difference-in-differences study based on 2016–2018 CFPS data. Int J Environ Res Public Health. (2022) 19:101. doi: 10.3390/ijerph19010101 PMC8749999 35010361

[19] Antonucci TC Ajrouch KJ Birditt KS. The convoy model: explaining social relations from a multidisciplinary perspective. Gerontologist. (2014) 54:82–92. doi: 10.1093/geront/gnt118, PMID: 24142914 PMC3894851

[20] Oreilly P. Methodological issues in social support and social network research. Soc Sci Med. (1988) 26:863–73. doi: 10.1016/0277-9536(88)90179-7 3287636

[21] Siette J Pomare C Dodds L Jorgensen M Harrigan N Georgiou A. A comprehensive overview of social network measures for older adults: a systematic review. Arch Gerontol Geriatr. (2021) 97:104525. doi: 10.1016/j.archger.2021.104525, PMID: 34536656

[22] Qi M Zhou SJ Guo ZC Zhang LG Min HJ Li XM. The effect of social support on mental health in Chinese adolescents during the outbreak of COVID-19. J Adolesc Health. (2020) 67:514–8. doi: 10.1016/j.jadohealth.2020.07.001, PMID: 32753347 PMC7395830

[23] Li B Li WT Zhong BL. Feelings of loneliness and mental health needs and services utilization among Chinese residents during the COVID-19 epidemic. Glob Health. (2021) 17:51. doi: 10.1186/s12992-021-00704-5, PMID: 33902638 PMC8072077

[24] Tomova L Wang KL Thompson T Matthews GA Takahashi A Tye KM. Acute social isolation evokes midbrain craving responses similar to hunger. Nat Neurosci. (2020) 23:1597–605. doi: 10.1038/s41593-020-00742-z, PMID: 33230328 PMC8580014

[25] Chopik WJ. Associations among relational values, support, health, and well-being across the adult lifespan. Pers Relat. (2017) 24:408–22. doi: 10.1111/pere.12187

[26] Antonucci TC Akiyama H. Social networks in adult life and a preliminary examination of the convoy model. J Gerontol. (1987) 42:519–27. doi: 10.1093/geronj/42.5.519, PMID: 3624811

[27] Due P Holstein B Lund R Modvig J Avlund K. Social relations: network, support and relational strain. Soc Sci Med. (1999) 48:661–73. doi: 10.1016/S0277-9536(98)00381-5 10080366

[28] Carstensen LL. Motivation for social contact across the life span: a theory of socioemotional selectivity In: Jacobs JE, editor. Nebraska symposium on motivation, 1992: Developmental perspectives on motivation. Lincoln, NE: University of Nebraska Press (1993). 209–54. 1340521

[29] Carstensen LL Isaacowitz DM Charles ST. Taking time seriously: a theory of socioemotional selectivity. Am Psychol. (1999) 54:165–81. doi: 10.1037/0003-066X.54.3.165, PMID: 10199217

[30] Zheng Z Chen H. Age sequences of the elderly’ social network and its efficacies on well-being: an urban-rural comparison in China. BMC Geriatr. (2020) 20:372. doi: 10.1186/s12877-020-01773-8, PMID: 32993525 PMC7526405

[31] Cacioppo S Grippo AJ London S Goossens L Cacioppo JT. Loneliness: clinical import and interventions. Perspect Psychol Sci. (2015) 10:238–49. doi: 10.1177/1745691615570616, PMID: 25866548 PMC4391342

[32] van Tilburg TG Steinmetz S Stolte E van der Roest H de Vries DH. Loneliness and mental health during the COVID-19 pandemic: a study among Dutch older adults. J Gerontol B Psychol Sci Soc Sci. (2021) 76:E249–55. doi: 10.1093/geronb/gbaa111, PMID: 32756931 PMC7454922

[33] Bahr HM. Loneliness: a sourcebook of current theory, research and therapy. Contemp Sociol. (1984) 13:203–4. doi: 10.2307/2068915

[34] Vo B. Networks in lockdown: the consequences of COVID-19 for social relationships and feelings of loneliness. Soc Networks. (2023) 72:1–12. doi: 10.1016/j.socnet.2022.08.001, PMID: 35968494 PMC9359936

[35] Heinrich LA Gullone E. The clinical significance of loneliness: a literature review. Clin Psychol Rev. (2006) 26:695–718. doi: 10.1016/j.cpr.2006.04.002, PMID: 16952717

[36] Bierman A Upenieks L Schieman S. Socially distant? Social network confidants, loneliness, and health during the COVID-19 pandemic. Soc Curr. (2021) 8:299–313. doi: 10.1177/23294965211011591

[37] Kovacs B Caplan N Grob S King M. Social networks and loneliness during the COVID-19 pandemic. Socius. (2021) 7:237802312098525. doi: 10.1177/2378023120985254

[38] Qian Z Li B Liao LY Liao GC Chen HJ Han JQ. Loneliness as a mediation from social support leading to a decrease of health-related quality of life among PLWHIV. Front Public Health. (2023) 10:1067870. doi: 10.3389/fpubh.2022.1067870, PMID: 36684920 PMC9846772

[39] Chopik WJ. The benefits of social technology use among older adults are mediated by reduced loneliness. Cyberpsychol Behav Soc Netw. (2016) 19:551–6. doi: 10.1089/cyber.2016.0151, PMID: 27541746 PMC5312603

[40] Morris ME Adair B Ozanne E Kurowski W Miller KJ Pearce AJ. Smart technologies to enhance social connectedness in older people who live at home. Australas J Ageing. (2014) 33:142–52. doi: 10.1111/ajag.12154, PMID: 24730370

[41] Katz JE Aspden P. A nation of strangers? *Commun*. ACM. (1997) 40:81–6. doi: 10.1145/265563.265575

[42] Mcmellon CA Schiffman LG. Cybersenior empowerment: how some older individuals are taking control of their lives. J Appl Gerontol. (2002) 21:157–75. doi: 10.1177/07364802021002002

[43] Nyqvist F Gustavsson JMC Gustafsson Y. Social capital and health in the oldest old: the Umeå 85+ study. Int. J. Ageing Later Life. (2006) 1:91–114. doi: 10.3384/ijal.1652-8670.061191

[44] Putnam RD. Bowling alone: The collapse and revival of American community. New York, NY: Touchstone Books/Simon & Schuster (2000).

[45] Nichols Dauner K Wilmot NA Schultz JF. Investigating the temporal relationship between individual-level social capital and health in fragile families. BMC Public Health. (2015) 15:1130. doi: 10.1186/s12889-015-2437-3, PMID: 26572491 PMC4647308

[46] Parks MR Floyd K. Making friends in cyberspace. J Comput-Mediat Comm. (1996) 1:601–14. doi: 10.1111/j.1083-6101.1996.tb00176.x

[47] Erickson L. B. (2011). Social media, social capital, and seniors: the impact of Facebook on bonding and bridging social capital of individuals over 65. In Americas Conference on Information Systems.

[48] Rheingold H. A slice of life in my virtual community In: Harasim LM, editor. Global networks: Computers and international communication. Cambridge, MA: MIT Press (1993). 57–80.

[49] Granovetter MS. The strength of weak ties. Am J Sociol. (1973) 78:1360–80. doi: 10.1086/225469

[50] Harasim LM. Global networks: Computers and international communication, vol. 19. Cambridge, MA: MIT Press (1994).

[51] Gardiner C Geldenhuys G Gott M. Interventions to reduce social isolation and loneliness among older people: an integrative review. Health Soc Care Community. (2018) 26:147–57. doi: 10.1111/hsc.12367, PMID: 27413007

[52] Shapira N Barak A Gal I. Promoting older adults’ well-being through internet training and use. Aging Ment Health. (2007) 11:477–84. doi: 10.1080/13607860601086546 17882585

[53] Furlong MS. An electronic Community for Older Adults: the SeniorNet network. J Commun. (2006) 39:145–53. doi: 10.1111/j.1460-2466.1989.tb01048.x

[54] Wright K. Computer-mediated social support, older adults, and coping. J Commun. (2000) 50:100–18. doi: 10.1111/j.1460-2466.2000.tb02855.x

[55] Radloff LS. The CES-D scale:a self-report depression scale for research in the general population. Appl Psychol Meas. (1977) 1:385–401. doi: 10.1177/014662167700100306

[56] Cong Z Silverstein M. Intergenerational time-for-money exchanges in rural China: does reciprocity reduce depressive symptoms of older grandparents? Res Hum Dev. (2008) 5:6–25. doi: 10.1080/15427600701853749

[57] Li Y Chan WCH Chen H Ran M. Widowhood and depression among Chinese older adults: examining coping styles and perceptions of aging as mediators and moderators. Aging Ment Health. (2022) 26:1161–9. doi: 10.1080/13607863.2021.1935455, PMID: 34121528

[58] Lubben J Blozik E Gillmann G Iliffe S von Renteln Kruse W Beck JC. Performance of an abbreviated version of the Lubben social network scale among three European Community-dwelling older adult populations. The Gerontologist. (2006) 46:503–13. doi: 10.1093/geront/46.4.503 16921004

[59] Kuru Alici N Kalanlar B. Validity and reliability of the Lubben social network scale-revised (LSNS-R) on older adults in Turkey. Curr Psychol. (2021) 40:21–8. doi: 10.1007/s12144-020-01125-0

[60] Yu K Wu S Chi I. Internet use and loneliness of older adults over time: the mediating effect of social contact. J Gerontol - B Psychol. (2020) 76:541–50. doi: 10.1093/geronb/gbaa004 31942629

[61] Minagawa Y Saito Y. An analysis of the impact of cell phone use on depressive symptoms among Japanese elders. Gerontology. (2014) 60:539–47. doi: 10.1159/000363059, PMID: 24968927

[62] Liu X Liu F Ruan W Chen Y Qu S Wang W. Mental health status and associated contributing factors among the Hakka elderly in Fujian, China. Front Public Health. (2022) 10:928880. doi: 10.3389/fpubh.2022.928880, PMID: 35937219 PMC9354451

[63] Wolters NE Mobach L Wuthrich VM Vonk P van der Heijde CM Wiers RW. Emotional and social loneliness and their unique links with social isolation, depression and anxiety. J Affect Disord. (2023) 329:207–17. doi: 10.1016/j.jad.2023.02.096, PMID: 36842647

[64] Yang H-L Zhang S Zhang S-Q Xie L Wu Y-Y Yao Y-D. Internet use and depressive symptoms among older adults in China. Front Psych. (2021) 12:739085. doi: 10.3389/fpsyt.2021.739085, PMID: 34950065 PMC8688754

[65] Carstensen LL. Socioemotional selectivity theory: the role of perceived endings in human motivation. The Gerontologist. (2021) 61:1188–96. doi: 10.1093/geront/gnab116, PMID: 34718558 PMC8599276

[66] Tang D Mair CA Hu Q. Widowhood, social networks, and mental health among Chinese older adults: the moderating effects of gender. Front Psychol. (2023) 14:1142036. doi: 10.3389/fpsyg.2023.1142036, PMID: 37077844 PMC10106722

[67] Hawkley LC Hughes ME Waite LJ Masi CM Thisted RA Cacioppo JT. From social structural factors to perceptions of relationship quality and loneliness: the Chicago health, aging, and social relations study. J Gerontol B Psychol Sci Soc Sci. (2008) 63:S375–84. doi: 10.1093/geronb/63.6.S375, PMID: 19092047 PMC2769562

[68] Tomaka J Thompson S Palacios R. The relation of social isolation, loneliness, and social support to disease outcomes among the elderly. J Aging Health. (2006) 18:359–84. doi: 10.1177/0898264305280993 16648391

[69] Victor C. Loneliness and social isolation among older Irish people. Ageing Society. (2006) 26:333–4. doi: 10.1017/S0144686x06274890

[70] Lifshitz R Nimrod G Bachner YG. Internet use and well-being in later life: a functional approach. Aging Ment Health. (2018) 22:85–91. doi: 10.1080/13607863.2016.1232370, PMID: 27657190

[71] Nowland R Necka EA Cacioppo JT. Loneliness and social internet use: pathways to reconnection in a digital world? Perspect Psychol Sci. (2018) 13:70–87. doi: 10.1177/1745691617713052, PMID: 28937910

[72] Allaire JC McLaughlin AC Trujillo A Whitlock LA LaPorte L Gandy M. Successful aging through digital games: socioemotional differences between older adult gamers and non-gamers. Comput Hum Behav. (2013) 29:1302–6. doi: 10.1016/j.chb.2013.01.014

[73] Zhou R Fong PS-W Tan P. Internet use and its impact on engagement in leisure activities in China. PLoS One. (2014) 9:e89598. doi: 10.1371/journal.pone.0089598 24586902 PMC3931801

[74] Nimrod G. The benefits of and constraints to participation in seniors’ online communities. Leis Stud. (2014) 33:247–66. doi: 10.1080/02614367.2012.697697

[75] Kamin ST Lang FR Kamber T. Social contexts of technology use in old age In: Kwon S, editor. Gerontechnology: Research, practice, and principles in the field of technology and aging. New York, NY: Springer Publishing Company (2017). 35–56.

[76] Russell C Campbell A Hughes I. Research: ageing, social capital and the internet: findings from an exploratory study of Australian ‘silver surfers’. Australas J Ageing. (2008) 27:78–82. doi: 10.1111/j.1741-6612.2008.00284.x, PMID: 18713197

[77] Neves B Fonseca J Amaro F Pasqualotti A. Social capital and internet use in an age-comparative perspective with a focus on later life. PLoS One. (2018) 13:e0192119. doi: 10.1371/journal.pone.0192119, PMID: 29481556 PMC5826529

[78] Antonucci TC Ajrouch KJ Manalel JA. Social relations and technology: continuity, context, and change. Innov Aging. (2017) 1:igx029. doi: 10.1093/geroni/igx029 29795794 PMC5954608

[79] Lang FR. Endings and continuity of social relationships: maximizing intrinsic benefits within personal networks when feeling near to death. J Soc Pers Relat. (2000) 17:155–82. doi: 10.1177/0265407500172001

[80] Cho J Smith J. Relocation later in life and contact frequency with friends: do contact modes matter? Res Aging. (2023) 45:486–97. doi: 10.1177/01640275221126103, PMID: 36112761 PMC10011020

